# The zhuyu pill relieves rat cholestasis by regulating the mRNA expression of lipid and bile metabolism associated genes

**DOI:** 10.3389/fphar.2023.1280864

**Published:** 2023-10-10

**Authors:** Jun Han, Peijie Wu, Yueqiang Wen, Chao Liu, Xinglong Liu, Huan Tao, Fenghua Zhang, Xiaodan Zhang, Qiaobo Ye, Tao Shen, Xiaofeng Chen, Han Yu

**Affiliations:** ^1^ School of Basic Medicine, Chengdu University of Traditional Chinese Medicine, Chengdu, China; ^2^ Department of Pediatrics, Guang’an Traditional Chinese Medicine Hospital, Guang’an, China; ^3^ Department of Dermatology, Cangxi Traditional Chinese Medicine Hospital, Guangyuan, China

**Keywords:** cholestasis, interventional mechanism, bile metabolism, lipid metabolism, zhuyu pill

## Abstract

**Background:** The Zhuyu pill (ZYP), composed of *Coptis chinensis* Franch. and *Tetradium ruticarpum* (A. Jussieu) T. G. Hartley, is an effective traditional Chinese medicine with potential anti-cholestatic effects. However, the underlying mechanisms of ZYP remain unknown.

**Objective:** To investigate the mechanism underlying the interventional effect of ZYP on mRNA-seq analysis in cholestasis rat models.

**Materials and methods:** This study tested the effects of a low-dose (0.6 g/kg) and high-dose (1.2 g/kg) of ZYP on a cholestasis rat model induced by α-naphthyl-isothiocyanate (ANIT, 50 mg/kg). Serum biochemistry and histopathology results were used to evaluate the therapeutic effect of ZYP, and mRNA-Seq analysis was performed and verified using real-time fluorescence quantitative PCR (qRT-PCR). GO, KEGG, and GSEA analyses were integrated to identify the mechanism by which ZYP impacted cholestatic rats.

**Results:** ZYP was shown to significantly improve abnormal changes in the biochemical blood indexes and liver histopathology of cholestasis rats and regulate pathways related to bile and lipid metabolism, including fatty acid metabolism, retinol metabolism, and steroid hormone biosynthesis, to alleviate inflammation, cholestasis, and lipid metabolism disorders. Relative expression of the essential genes Cyp2a1, Ephx2, Acox2, Cyp1a2, Cyp2c11, and Sult2a1 was verified by qRT-PCR and showed the same trend as mRNA-seq analysis.

**Conclusion:** ZYP has a significant anti-cholestatic effect by regulating bile metabolism and lipid metabolism related pathways. These findings indicate that ZYP is a novel and promising prospect for treating cholestasis.

## Introduction

In cholestasis, bile production, secretion, and excretion are blocked. Bile can no longer flow into the duodenum and enters the bloodstream instead. When the disease progresses, hyperbilirubinemia can occur, which, in severe cases, leads to cirrhosis, liver failure, or even death (Wu et al., 2021). Primary biliary cirrhosis (PBC) and primary sclerosing cholangitis (PSC) are the most acute cholestatic liver diseases. 882 (35%) of 2,520 patients with initially diagnosed chronic liver disease had cholestasis, which was more prevalent in PBC and PSC (Bortolini et al., 1992). A study of 1,000 patients with chronic viral hepatitis showed that 56% were discharged with ALP or GGT above the upper limit of normal (ULN), for whom disease severity and the risk of liver fibrosis and cirrhosis were significantly increased (Xie et al., 2017). Ursodeoxycholic acid (UDCA) is the most common drug used to treat PBC. However, some PBC patients do not respond well to UDCA, and no effective treatment has been developed for PSC (Lu, 2022). Cholestasis has become a public health problem of general concern to medical researchers due to its adverse outcomes and the absence of reliable treatment options. The design of effective drugs is urgently needed in clinical practice.

Traditional Chinese medicine (TCM) has a unique understanding of the pathogenesis and treatment of cholestasis. From the TCM perspective, cholestasis is primarily related to poor liver function and biliary *qi*, which are accompanied by the accumulation of pathological products. Based on this concept, *Coptis Chinensis* Franch. and *Tetradium ruticarpum* (A. Jussieu) T. G. Hartley, which can regulate the movement of *qi* in the liver and bile, are still widely used in the clinical treatment of digestive diseases.

Previous studies (Ma and Ma, 2013; Wang, 2016; Liu et al., 2017) have shown that *Coptis Chinensis* Franch. has anti-inflammatory, anti-hepatic steatosis, anti-oxidant, anti-tumor, anti-diabetic, anti-arrhythmic, and anti-hypertensive effects while *T. ruticarpum* (A. Jussieu) T. G. Hartley has analgesic, anti-inflammatory, anti-tumor, and anti-oxidant effects on the cardiovascular, central nervous, digestive, reproductive, and other biological systems (Yang et al., 2011; Du and Yao, 2013; Liu et al., 2016; Wu and Chen, 2019; Zhu et al., 2019; Xu et al., 2021). The combination of these botanical drugs (commonly known as the Coptis-Evodia botanical drug couple, CEBC) is effective against various digestive disorders by reducing the accumulation of fat in the liver and protecting liver function through various pathways, including reducing FGF21 secretion, upregulating ABCA1 mRNA expression, promoting reverse cholesterol transport, and upregulating GATA-2 and GATA-3 gene and protein expression (Shen, 2007; Hu et al., 2010; Zhang et al., 2022b). In addition, these botanical drugs have been shown to have significant lipid-lowering effects. (Shen, 2007; Shen et al., 2011).

Zhuyu pill (ZYP, representative formulae of CEHC, usually mixed at a 1:1 g/g ratio) was first documented in the official medical dictionary “*Tai Ping Sheng Hui Fang*” of the Song Dynasty and is now included in the “Prescription Dictionary of Chinese Medicine” (Peng, 1993), an officially recognized work in China. In traditional Chinese medicine theory, liver and biliary *qi* is a generalization of the liver and gallbladder functions, and liver and biliary *qi* dysfunction often contributes to digestive system disease. Zhuyu pill was traditionally used to treat hepatobiliary and gastrointestinal diseases for its prominent effect of improving the liver and biliary *qi* (Li, 2002). Previous studies (Yu et al., 2022) have shown that ZYP has a significant anti-cholestasis effect achieved through the dual effects of regulating fecal metabolic homeostasis and fecal microbial abundance, as well as regulating the expression of miRNAs such as miR-147 and its target genes in the liver. However, the mechanism by which ZYP treats cholestasis has not yet been fully characterized. Messenger RNA (mRNA) is a class of single-stranded ribonucleic acid transcribed from DNA and direct protein synthesis. In cholestasis, abnormal expression of transporters associated with bile acid metabolism and mRNA associated with lipogenesis and oxidative lipid metabolism in bile stasis (Qiu et al., 2021). This suggests that ZYP may play an anti-cholestasis role by modulating mRNA expression reverse these conditions. To confirm this hypothesis, cholestatic rats were treated with ZYP. The efficacy of this drug was evaluated by quantifying the levels of serum biochemical markers and assessing liver tissue pathology. Relevant metabolic pathways and differentially expressed mRNAs were screened by transcriptome sequencing based on a pharmacodynamic evaluation. This study sought to define further the mechanism for ZYP treatment of cholestasis using modern biological techniques, providing a biological basis for treating cholestasis using TCM.

## Materials and methods

### Reagent preparation

All the Chinese botanical drugs used in this experiment were purchased from Beijing Tongrentang Co., Ltd. (Beijing, China). Pentobarbital sodium, UDCA, and *α*-naphthylisothiocyanate (ANIT) were purchased from Sigma-Aldrich Co. (St. Louis, MO, United States). Olive oil was chosen from Shanghai Yi En Chemical Technology Co., Ltd. (Shanghai, China). The chemical reagents used in this experiment were all of high-performance liquid chromatography (HPLC) analytical grade.

### Preparation of ZYP and high performance liquid chromatography (HPLC) analysis

In Table 1, the characteristics of the two constituent botanical drugs are listed. ZYP Preparation adheres to the Science of Prescription guidelines as outlined in the Ministry of Education’s General Higher Education “13th Five Year Plan” national planning materials, using the traditional method of boiling Chinese herbal medicines, the dried botanical drugs, including *Coptis Chinensis* Franch. and *T. ruticarpum* (A. Jussieu) T. G. Hartley at the ratio of 1:1 (w/w), were immersed in purified water of 20 - fold volumes of botanical drugs (v/w) for 30 min and then were heated to boiling and were kept 30 min. The liquid was then filtered and collected. The decoction was boiled again as described above, and the liquid was collected, mixed with the initial liquid, concentrated to 120 mL. After filtration, the solution was evaporated under reduced pressure to a suspension with a final density of 0.1 g/mL, and stored at −20°C for backup (Li and Lian, 2016).

**TABLE 1:** Characteristics of the two constituent botanical drugs in Zhuyu pill.

Chinese name	Botanical name^a^	Genus family	Batch number	Medicinal parts	Origin	Weight (g)
Huanglian	*Coptis chinensis* Franch	Ranunculaceae	220701	Dried root	Chongqing, China	6
Wuzhuyu	*Tetradium ruticarpum* (A. Jussieu) T. G. Hartley	Rutaceae	220416008	Dried mature seed	Guizhou, China	6

^a^ The plant name was verified using http://www.theplantlist.org.

The aforementioned solution was subjected to reflux extraction twice, each time for a duration of 1 h, and this process was repeated three times. Subsequently, the extraction solution was concentrated under reduced pressure and freeze-dried. The extraction rate was determined to be 10.6% using acid dye colorimetry, indicating a drug extract ratio of 10.6 g per 100 g. To determine the main metabolites, four alkaloids (berberine, coptisine, evodiamine, and rutecarpine) in ZYP were analyzed via HPLC using an Agilent 1,260 Infinity II (Agilent Technologies Inc., California, United States). Chromatographic separation was performed with a Welch Ultimate XB-C18 Column (4.6 mm × 250 mm, 5 μm, Maryland, California, United States) at a column temperature of 30°C. The linear-gradient mobile phase consisted of mobile phase A (50 mM monopotassium phosphate +0.4% sodium heptane sulfonate, pH = 4) and mobile phase B (pure methanol). A mobile phase gradient was used (0–15 min, 95% A, 5% B; 15–40 min, 50% A, 50% B; 40–55 min, 30% A, 70% B; 55–60 min, 95% A, 5% B), with a 1.0 mL/min flow rate and 10ul injection volume. The detection wavelength was set as (0–44 min, 345 nm; 44–48 min, 226 nm; 48–60 min, 345 nm) (Yu et al., 2022). ZYP was found to contain 36.8 mg/g berberine, 14.9 mg/g coptisine, 0.78 mg/g evodiamine, and 0.33 mg/g rutecarpine (Yu et al., 2022) **(Supplementary Material 1)**.

### Animals and treatments

The experiments were conducted according to the internationally recognized Guiding Principles for the Care and Use of Laboratory Animals and the study received approval from the Animal Ethics Committee at the Chengdu University of Traditional Chinese Medicine. The ethics approval number for the use of animals in this study was 2019-15.

A total of 30 healthy male Sprague Dawley (SD) rats weighing 160–180 g were purchased from Beijing Harvest Biotechnology Co., Ltd. (Beijing, China; certification number: SCXK-JING, 2019-0008). After 4 days of acclimatization feeding, we randomly divided all rats into five groups: Control group (Control), Model group (Model), ZYP low-dose group (ZYP_L), ZYP high-dose group (ZYP_H), and Ursodeoxycholic acid group (UDCA), six rats in each group. The animal administration dosage of ZYP was determined to be 1.2 g/kg, taking into account the pre-experiment data and conversion based on body surface area. This dosage corresponds to the typical clinical dose of 12 g/60 kg. Beginning on day 5, rats in the ZYP_L, ZYP_H, and UDCA groups received daily administrations of 0.6 g/kg, 1.2 g/kg ZYP, and 60 mg/kg UDCA, respectively, by oral gavage until day 10. Meanwhile, rats in the Model and Control groups were given an equal volume of purified water. On day 11, the experimental groups were administered 50 mg/kg ANIT solution dissolved in olive oil. The Control rats were given the corresponding dose of olive oil, referring to the modeling method described previously (Xu and Miao, 2021). On days 12–14, rats in the ZYP_L, ZYP_H, and UDCA groups were given 0.6 g/kg, 1.2 g/kg of ZYP, and 60 mg/kg UDCA by gavage, respectively, while those in the Model and Control groups were given an equal volume of purified water. On day 15, all rats were sacrificed with 150 mg/kg sodium pentobarbital anesthesia, and blood and liver tissues were collected from each group for testing.

### Liver function assays

After fasting for 12 h, the rats were anesthetized using an intraperitoneal injection of sodium pentobarbital solution, blood was removed from the inferior vena cava, and the livers were harvested. Serum samples were obtained by centrifugation of blood samples at 3,500 × g for 15 min at 4°C. The relevant biochemical parameters, including ALT, AST, ALP, γ-GT, DBIL, TBIL, TBA, TC, and TG, were detected by a fully automated biochemical analyzer (BS-240VET). Liver tissues from each group of rats were fixed in 4% paraformaldehyde, rinsed with running water, dehydrated, embedded, sectioned, and HE stained. The stained tissues were examined microscopically, and images were acquired for analysis.

### RNA extraction and library construction

In each group, three liver tissue samples were randomly selected for mRNA sequencing. Total RNA was extracted using the mir Vana miRNA Isolation Kit (Ambion) according to the manufacturer’s protocol. RNA integrity was evaluated using the Agilent 2,100 Bioanalyzer (Agilent Technologies, Santa Clara, CA, United States). Samples with RNA Integrity Number (RIN) ≥7 were used for subsequent analysis. The libraries were constructed using TruSeq Stranded Total RNA with Ribo-Zero Gold according to the manufacturer’s instructions. The libraries were then sequenced on an Illumina sequencing platform, and 150/125 bp paired-end reads were generated.

### Bioinformatic analysis

Raw reads generated during high-throughput sequencing were fastq format sequences that required further quality filtering to obtain high-quality reads for later analysis. Trimmomatic software was used for adapter removal, and the low-quality bases and N-bases or low-quality reads were filtered out to get high-quality clean reads. Using hisat2 to align the clean reads to the reference genome of the experimental species, the sample was assessed by genomic and gene alignment. The alignment result with the reference genome was stored in a binary bam file, and the new transcript was spliced using Stringtie software to assemble the reads. The mRNA transcript sequences were aligned with the sequencing reads of each sample and eXpress was used to obtain the fragments per kilobase of transcript per million (FPKM) and count values (the number of reads per gene in each sample).

The estimateSizeFactors function of the DESeq (2012) R package was used to normalize the counts, and the nbinomTest function was used to calculate the *p*-value and fold change values for the difference comparison. Differential transcripts with *p*-values ≤0.05 and fold change ≥2 were selected, and differential mRNA GO and KEGG enrichment values were assessed using the Hypergeometric Distribution Test. mRNA sequencing and analysis were conducted using OE Biotech Co., Ltd. (Shanghai, China).

### mRNA validation using real-time quantitative qRT-PCR

The qRT-PCR verification of mRNA is generally divided into three steps: mRNA extraction, reverse transcription, and PCR quantification. The details of the specific experimental process in this part can be checked in **(Supplementary Material 2)**. The qRT-PCR primers (Table 2) used in this study were designed according to the mRNA sequences from the NCBI database and synthesized by TsingKe Biotech.

**TABLE 2:** qRT-PCR primers used in this study.

Gene symbol	Forward primer (5′–3′)	Reverse primer (5′–3′)	Product length (bp)	Tm (°C)
ACTB	GCG​AGT​ACA​ACC​TTC​TTG​C	TAT​CGT​CAT​CCA​TGG​CGA​AC	72	60
Alox15	CAA​CTG​GAA​GGA​TGG​CTC​A	TCC​TCT​CGA​AAT​CGT​TGG​T	81	60
Cyp2a1	ATG​GCA​ATT​CAG​AGT​TCC​AC	GAG​CTG​ACT​GTC​TCA​GAC​C	82	60
Ephx2	GCTGGACGACAGTGACAA	CGA​CCT​GAC​AGG​ACT​CTA​T	92	60
Acox2	TGC​CAT​GAA​TGC​TAT​CCG​A	TGTCTGGGCGTATGTTGT	100	60
Sult2a1	CAG​ATG​AGC​TGG​ATT​TGG​TC	CAT​GAG​GCC​AAT​TCC​AGT​AA	116	60
Cyp1a2	TGT​CAC​CTC​AGG​GAA​TGC​T	GAC​CAC​CGT​TGT​CTT​TGT​AG	212	60
Cyp2c11	ACG​TGG​ATG​TCA​CAG​CTA​AAG​TCC	GGC​TCC​GGT​TTC​TGC​CAA​TTA​C	63	60

### Statistical analysis

GraphPad Prism version 8 was used to assess the differences in serum biochemical indicators. One-way ANOVA was used for intergroup comparison. Statistical differences between the groups were assessed using the mean ± standard deviation, and *p* < 0.05 indicated that the differences were statistically significant.

## Results

### Impact of ZYP on liver function

**FIGURE 1:**
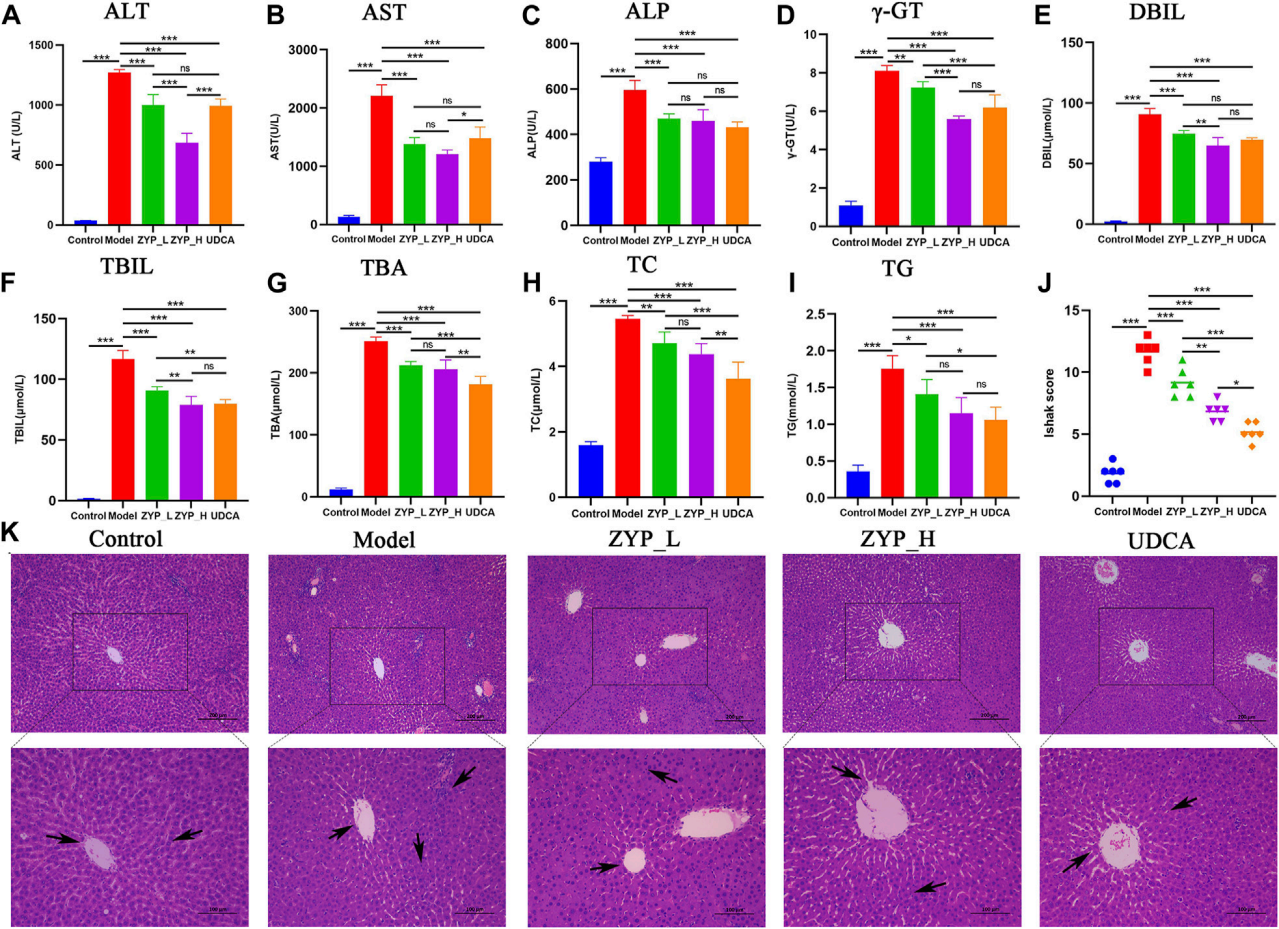
Serum biochemical tests and histopathological alterations of the liver in each group. **(A)** ALT, **(B)** AST, **(C)** ALP, **(D)** γ-GT, **(E)** DBIL, **(F)** TBIL, **(G)** TBA, **(H)** TC, **(I)** TG, **(J)** Ishak score of hepatic inflammation and necrosis, *n* = 6, ***, *p* < 0.001, **, *p* < 0.01, *, *p* < 0.05, ns, not significant. **(K)** Histopathological examination of each group at 100x and 200x.

As shown in Figures 1A−I, the serum levels of ALT, AST, ALP, γ-GT, DBIL, TBIL, TBA, TC, and TG in the rats in the cholestasis Model group were significantly higher than those in the Control group, while both ZYP_L and ZYP_H could significantly reduce the above indexes, showing a similar trend of action with UDCA. Moreover, the effect of ZYP in the treatment of cholestasis was a dose-effect relationship. While the liver tissue in the Control group showed a clear structure, tightly arranged cells with clear boundaries, abundant cytoplasm, uniform color, round nuclei, regular size, and an intact and normal venous endothelium, liver tissue in the Model group showed focal necrosis of hepatocytes and inflammatory cell infiltration. In the ZYP_L and ZYP_H groups, these pathological changes were improved to different degrees (Figure 1K). Combined with Ishak score analysis (Figure 1J), ZYP_H achieved a similar intervention effect as UDCA. These results indicate that ZYP has a positive therapeutic effect on cholestasis, especially when given at a higher dose. Therefore, the potential mechanism of ZYP_H for treating cholestasis is more valuable to investigate.

### Screening and quantitative statistics of differentially expressed mRNAs

DESeq software was used to normalize the counts of each sample mRNA (Base Mean value was used to estimate the expression), calculate the difference ploidy, test the different significance of counts by negative binomial (NB) distribution, and screen for differences in gene expression based on the ploidy and significance test results.

**FIGURE 2:**
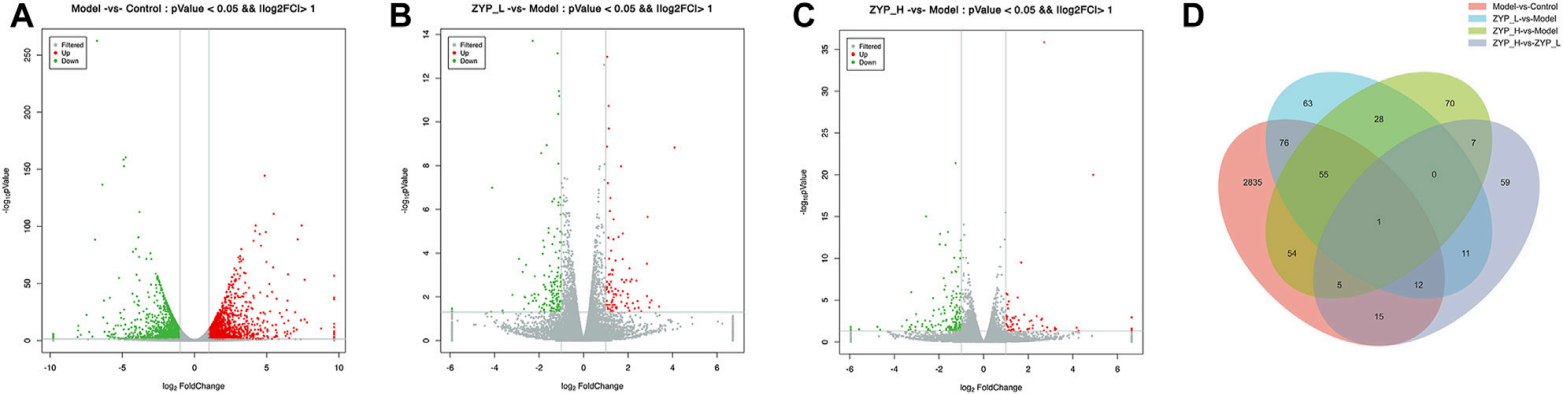
Differential gene expression analysis of the mRNA-Seq. **(A–C)** Volcano plot reflecting differences in gene expression. Genes with non-significant differences are gray, and genes with significant differences are red and green; **(D)** Numbers of the overlapping genes in different comparisons.

The screening criteria for significantly differentially expressed mRNAs were *p* < 0.05 and FC > 2. Figure 2 shows that 3,053 genes were significantly altered (1,653 upregulated and 1,400 downregulated) after ANIT induction, indicating that ANIT significantly altered gene expression in rat liver tissues. In contrast, both low-doses (93 upregulated and 153 downregulated) and high-doses (62 upregulated and 158 downregulated) of ZYP significantly changed the gene expression in the liver of cholestatic rats. These results indicated that ZYP treatment could reverse this expression trend, and these differentially expressed genes may be the regulatory targets of ZYP_H in treating cholestasis.

**FIGURE 3:**
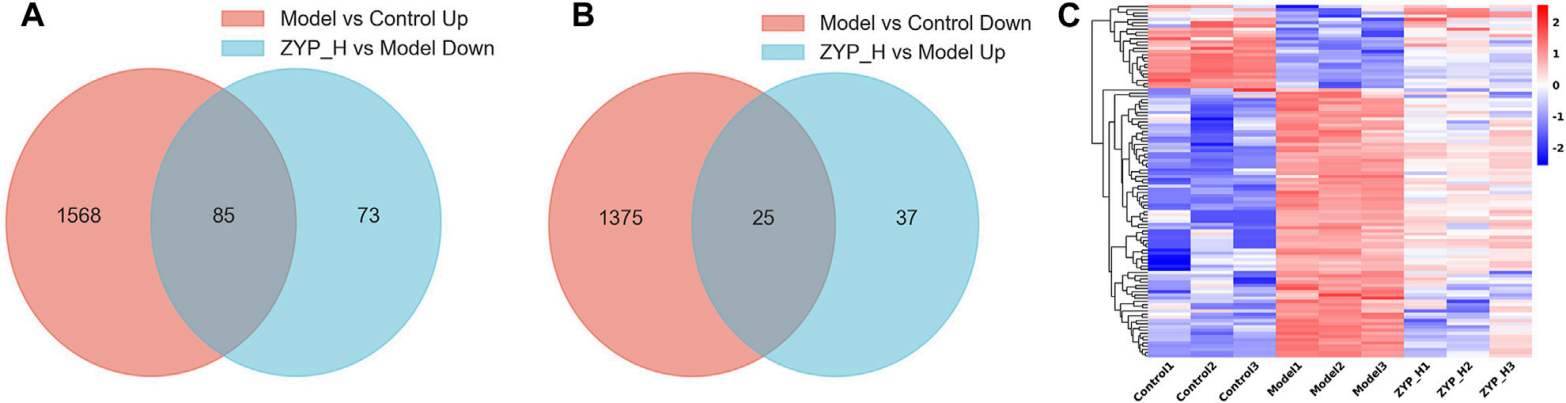
Target genes regulated by ZYP_H for the treatment of cholestasis. **(A)** Downregulated target genes of ZYP_H; **(B)** Upregulated target genes of ZYP_H; **(C)** Heatmap for hierarchical cluster analysis of different express genes between Control, Model, and ZYP_H.

To further elucidate the mode of action of ZYP in cholestasis, we focused on the target genes in treating cholestasis with ZYP_H. These target genes were divided into upregulated target genes and downregulated target genes. Compared to controls, genes that are downregulated in the cholestasis model and upregulated after ZYP_H treatment are upregulated target genes. Compared to the Control group, genes that were upregulated in the cholestasis model and downregulated after ZYP_H treatment were downregulated target genes. The present study showed 85 downregulated target genes and 25 upregulated target genes in ZYP_H treating cholestasis (Figures 3A, B). The expression of these target genes significantly differed between groups. More notably, the ANIT-induced cholestasis model, which had abnormal gene expression, showed a similar gene expression trend to the Control group after the ZYP_H intervention (Figure 3C). This indicates that ZYP_H may reverse-regulate the abnormal expression of genes caused by ANIT.

### Functional description and pathway analysis of differentially expressed mRNAs

**FIGURE 4:**
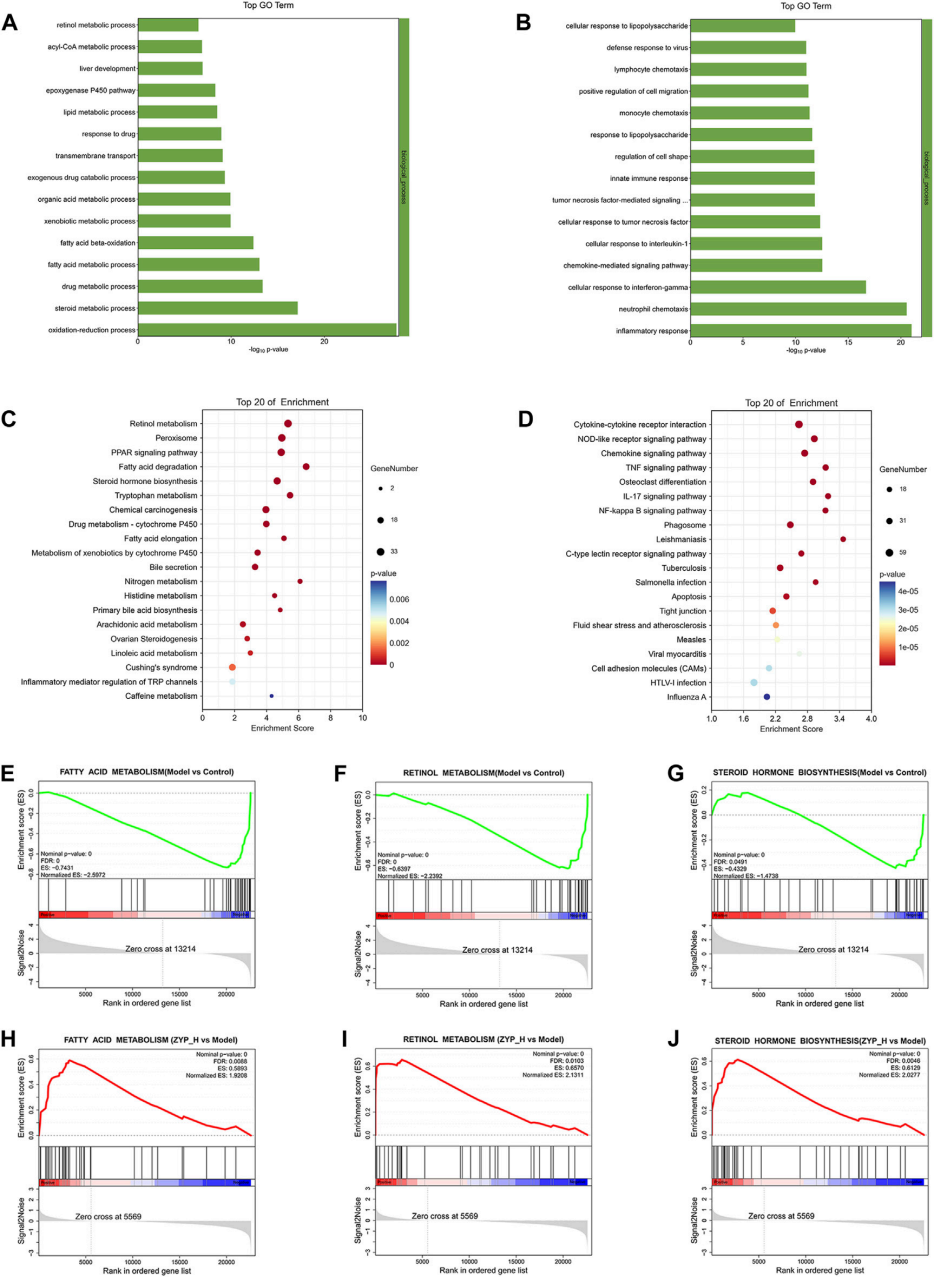
Functional analysis of differentially expressed mRNAs. **(A, C)** Biological processes and signaling pathways inhibited in the Model group and promoted after ZYP_H treatment; **(B, D)** Biological processes and signaling pathways promoted in the Model group and inhibited after ZYP_H treatment. **(E–J)**, GSEA analysis of the differentially expressed mRNAs between Model vs Control-down and ZYP_H vs Model-up, **(E, H)** fatty acid metabolism; **(F, I)** retinol metabolism; **(G, J)** steroid hormone biosynthesis.

To determine the function of target genes regulated by ZHP_H during the treatment of cholestasis, GO and KEGG analyses were performed on differentially expressed mRNAs (Figures 4A–D). Compared to the Control group, biological processes and signaling pathways inhibited in the Model group and promoted after ZYP_H treatment, and those promoted in the Model group and inhibited after ZYP_H treatment were the therapeutic targets of ZYP_H in cholestasis.

GO enrichment analysis showed that the biological processes upregulated by ZYP_H were mainly involved in the steroid metabolic process, fatty acid metabolic process, lipid metabolic process, epoxygenase P450 pathway, and retinol metabolism. In contrast, the biological processes downregulated by the ZYP_H group included inflammatory response, neutrophil chemotaxis, cellular response to interleukin-1, etc. Combined with KEGG analysis, ZYP_H upregulated signaling pathways involved retinol metabolism, fatty acid degradation, arachidonic acid metabolism, steroid hormone biosynthesis, PPAR signaling pathway, and bile secretion. ZYP_H downregulated signaling pathways included cytokine-cytokine receptor interaction, IL-17 signaling pathway, Chemokine signaling pathway, etc. This was consistent with the results of the GO analysis. In general, the biological processes and signaling pathways promoted by ZYP_H in the treatment of cholestasis were mainly related to lipid metabolism and bile metabolism, while the biological processes and signaling pathways inhibited by ZYP_H were mainly related to inflammatory response and immune response. Considering the pathological process of bile secretion and excretion disorders caused by cholestasis, the bile metabolism and lipid metabolism-related pathways regulated by ZYP_H have become the focus of our research.

In addition, Gene Set Enrichment analysis (GSEA) was performed on all differentially expressed genes to avoid screening out essential genes with weak changes resulting from the fixed threshold screening method (Figures 4E–J). The results likewise indicated that fatty acid metabolism, retinol metabolism, and steroid hormone biosynthesis were upregulated target pathways of ZYP _H. These data suggested that ZYP has a therapeutic effect on cholestasis by regulating the expression of genes involved in lipid and bile metabolism.

### Regulatory mechanism of ZYP in treating cholestasis

**FIGURE 5:**
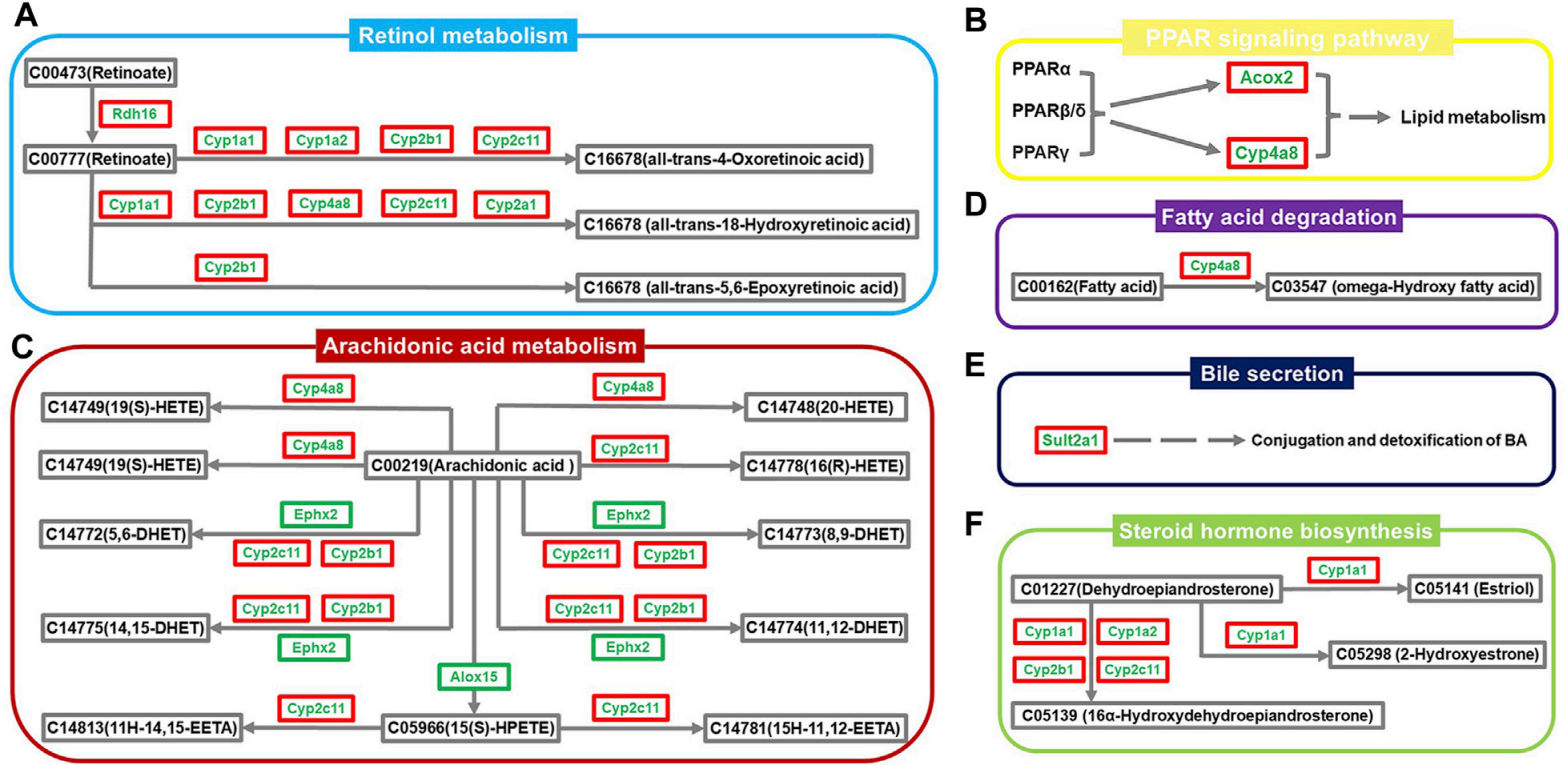
Network diagram of the regulatory mechanism of ZYP for cholestasis. **(A)** Retinol metabolism, **(B)** PPAR signaling pathway, **(C)** Arachidonic acid metabolism, **(D)** Fatty acid degradation, **(E)** Bile secretion, **(F)** Steroid hormone biosynthesis. Green names indicate that the gene is downregulated in the Model group compared to the Control group. The red outer box indicates that the gene is upregulated in the ZYP_H group compared to the Model group. The green outer box indicates that the gene is downregulated in the ZYP_H group compared to the Model group.

A regulatory network map was created using the above results to elaborate in more detail on the biological mechanisms underlying the therapeutic effects of ZYP on cholestasis. As shown in Figure 5, the pathways were inhibited to different degrees in the cholestasis model, and ZYP acted distinctly on each one. The genes regulated in all pathways, Cyp1a1, Cyp1a2, Cyp2a1, Cyp2b1, Cyp4a8, Cyp2c11, Rdh16, Alox15, Ephx2, Sult2a1, and Acox2, were the potential targets for ZYP treatment of cholestasis.

### qRT-PCR validation of mRNA-Seq and correlation analysis between core genes and serum biochemistry

**FIGURE 6:**
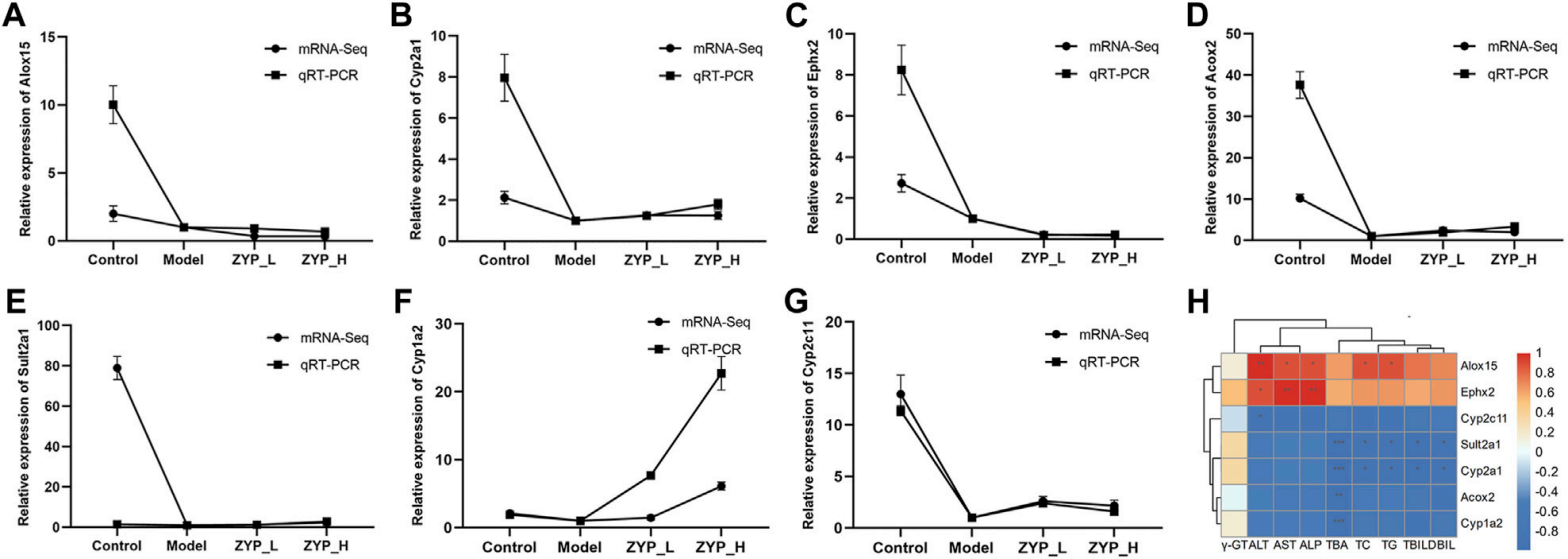
qRT-PCR validation of mRNA-Seq and correlation analysis between core genes and serum biochemistry. The genes selected for qRT-PCR validation were enriched in the regulatory network diagram in Figure 5. **(A)** Alox15, **(B)** Cyp2a1, **(C)** Ephx2, **(D)** Acox2, **(E)** Sult2a1, **(F)** Cyp1a2, **(G)** Cyp2c11. Data are expressed as the means ± sd (*n* = 3). **(H)** Correlation analysis between core genes and serum biochemistry: Orange-red is positive, blue is negative, and darker color indicates stronger correlation. *, *p* < 0.05, **, *p* < 0.01, ***, *p* < 0.001 (This result was done with Oebiotech cloud platform tools at the following website: https://cloud.oebiotech.cn/task/).

To determine the accuracy and reliability of mRNA-seq, several core genes, Alox15, Cyp2a1, Ephx2, Acox2, Sult2a1, Cyp1a2, and Cyp2c11 were validated by qRT-PCR (Figure 6). The expression trends of all genes except Alox15 were consistent with mRNA-seq, indicating that the findings were reliable. Spearman’s calculation method analyzed the correlation of core gene expression differences with ZYP_H for cholestasis. The findings of this study indicate that the expression levels of Sult2a1 and Cyp2a1 were found to have a significant negative correlation with TC, TG, TBA, TBIL, and DBIL. Acox2 and Cyp1a2 were significantly and negatively correlated with TBA, and Cyp2c11 negatively correlated with ALT. Conversely, the expression of Alox15 was significantly and positively correlated with ALT, AST, ALP, TC, and TG. Furthermore, the expression of Ephx2 was significantly and positively correlated with AST, ALT, and ALP. These results suggest that ZYP_H may have the potential to ameliorate cholestasis and enhance lipid metabolism by modulating the expressions of Alox15, Acox2, Cyp2a1, and Sult2a1, while also improving liver function.

## Discussion

ANIT is an indirect hepatotoxic agent that damages intrahepatic bile duct epithelial cells, causing capillary hyperplasia and inflammation around the interlobular bile ducts, eventually leading to bile duct epithelial necrosis and obstruction by shedding. This results in evident bile excretion disorder accompanied by parenchymal cell damage through punctate necrosis, producing biliary stasis (Labiano et al., 2022). TCM theory considers that cholestasis requires the pungent-opening and bitter-subduing method to improve the movement of *qi*, and ZYP is a classic formula for this therapy. Serum biochemistry and pathological observations are the fundamental indicators of cholestasis, liver injury, and efficacy evaluation. ALT, AST, ALP, γ-GT, TBA, DBIL, TBIL, TG and TC levels can objectively and effectively reflect liver function, cholestasis, and lipid metabolism (Fickert et al., 2017; Nong et al., 2020). Pathological observation is the gold standard for diagnosis and is essential for determining the extent of liver damage. This study confirmed that ZYP had a dose-dependent therapeutic effect on cholestasis by pathological observations and serum biochemical indexes, which is consistent with our previous work (Yu et al., 2022). mRNA transcriptome sequencing and the ZYP gene regulation map identified six pathways and five differential genes that may be involved in ZYP’s mechanism of action against cholestasis.

Cholestasis is usually associated with impaired fatty acid metabolism in the liver, and cholestatic and non-alcoholic fatty liver disease share several fundamental pathophysiological mechanisms (Trauner and Fuchs, 2022). Inflammation is also a critical pathological factor in cholestasis. The glucocorticoids in steroids inhibit the release of inflammatory factors, promote bile excretion, and reduce impaired liver function (Yan et al., 2016).

Steroid hormone biosynthesis also promotes cholesterol conversion and bile acid synthesis, increasing cholesterol excretion from the liver (Sun et al., 2021). The current study found that retinol metabolism was significantly inhibited in the livers of rats in the cholestasis model, which supports previous studies (Cai et al., 2014; Takitani et al., 2018; Yuan et al., 2018). Retinoic acid may improve lipid deposition. Retinoic acid upregulates peroxisome proliferator-activated receptor alpha (PPAR-α) and retinoic acid-like receptor alpha (RXR-α) to promote fatty acid oxidation and inhibits fatty acid synthesis via SREBP-1c and fatty acid synthase (Senoo et al., 2017; Cassim Bawa et al., 2022). Lipotoxicity caused by fatty acid accumulation can induce stress in hepatocytes and bile duct cells (Natarajan et al., 2017). Thus, increasing fatty acid metabolism and degradation can help to prevent lipid metabolism disorder and cholestasis.

Bile secretion, steroid hormone biosynthesis, and the PPAR signaling pathway play an essential role in regulating cholesterol and bile acid homeostasis by affecting biliary secretion and reducing the inflammatory response. Acox2 and Sult2a1 are essential genes in PPAR signaling and bile secretion, and studies have revealed a correlation between the expression of these genes and cholestasis. Acox2 is involved in bile acid biosynthesis, particularly in regulating bile acid intermediate metabolism and branched-chain fatty acid oxidation (Zhang et al., 2022a). Acox2 deficiency is characterized by the accumulation of bile acids and intermediates (Monte et al., 2017), bile acid synthesis, and elevated transaminase production (Alonso-Peña et al., 2022). PPARα regulates bile acid detoxification by upregulating Sult2a1 (Ghonem et al., 2015), which plays an essential role in catalyzing the sulfation of bile acids and promoting the elimination of toxic secondary bile acids (Kong et al., 2021), thereby alleviating bile stasis. ZYP upregulates the expression of Acox2 and Sult2a1 to induce bile metabolism and reduce liver injury.

Arachidonic acid is a critical inflammatory mediator that regulates oxidative stress and mediates hepatocyte injury. Alox15 and Ephx2 are potential hub genes in bile acid metabolism, which correlates with liver function. The downregulation of these genes is shown to improve liver injury, inflammation, and steatosis (Martínez-Clemente et al., 2010; Mello et al., 2021) and promote drug and fatty acid metabolism, thereby reducing liver toxicity (Zhang et al., 2017; Almansour et al., 2018). The current study showed that Alox15 and Ephx2 expression were downregulated by ZYP, suggesting that this drug may inhibit inflammation and improve liver function. However, it is worth noting that these two genes were also downregulated in the Model and Control groups, suggesting that they are not the anti-cholestasis targets of ZYP. Further research is required to determine their precise mechanisms of action.

Cyp enzymes also play a pivotal role in the biotransformation of steroids, fatty acids, and bile acids. Surprisingly, almost all cholestasis-specific pathways regulated by ZYP involve Cyp enzymes. Prior studies indicate that Cyp1a1, Cyp1a2, and Cyp2b1 are potential targets for treating of cholestasis (Ding et al., 2014; Ibrahim, 2015; Wang et al., 2022). Cyp2c11 exhibits significant catalytic activity in the metabolism of arachidonic acid, but its activity is diminished in various inflammation models, leading to lipid accumulation and liver injury (Sugatani et al., 2006; Zordoky et al., 2011). The expression and function of Cyp1a2 are diminished in cases of inflammation and cholestasis, which are both linked to the development of steatosis and cholestasis. (Klein et al., 2010; Deng et al., 2023). Our experiments showed that ZYP has a significant inverse regulatory effect on the expression of these genes. Cyp2a1 was confirmed as a critical enzyme in melatonin metabolism, helping to protect the bromoamide derivatives of melatonin from metabolic effects (Sangchart et al., 2021). Interestingly, both endogenous and exogenous melatonin can ameliorate liver injury by reducing oxidative stress, inflammatory response, and biliary senescence (Hu et al., 2009; Wu et al., 2017; Yu et al., 2018). The current study found that ZYP significantly increased the expression of Cyp2a1 in the liver of cholestatic rats, suggesting that promoting endogenous melatonin secretion is a potential mechanism for the anti-cholestatic effect of ZYP.

Finally, analysis of core gene expression and serum biochemistry results indicated that the promotion of bile acid and lipid catabolism and detoxification by ZYP was primarily associated with the upregulation of Sult2a1, Cyp2a1, Cyp1a2, and Acox2. In contrast, the improvement in liver function was mainly achieved through the downregulation of Cyp2c11, Alox15 and Ephx2. However, γ-GT levels were not significantly correlated with serum biochemistry results, which is consistent with our previous results (Yu et al., 2021). Future experiments are needed to further investigate this finding.

## Conclusion

ZYP was found to have a significant dose-response effect on cholestasis, related to its regulation of the expression of mRNAs related to bile and lipid metabolism. The findings from this study contribute to the “TCM wisdom” used to diagnose and treat this disease.

## Data Availability

The datasets presented in this study can be found in online repositories. The names of the repository/repositories and accession number(s) can be found below: NCBI BioProject (https://www.ncbi.nlm.nih.gov/bioproject/), PRJNA908063

## Abbreviations

ANIT, *α*-naphthylisothiocyanate; GO, Gene ontology; HE, Hematoxylin-eosin; HPLC, High-performance liquid chromatography; KEGG, Kyoto Encyclopedia of Genes and Genomes; qRT-PCR, Real-time fluorescence quantitative PCR; UDCA, Ursodeoxycholic acid; ALT, Alanine aminotransferase; *γ*-GT, *γ*-glutamyl transpeptidase; TC, Total cholesterol; TBATotal bile acid.

## References

[B1] Almansour M. I. Jarrar Y. B. Jarrar B. M. (2018). *In vivo* investigation on the chronic hepatotoxicity induced by sertraline. Environ. Toxicol. Pharmacol. 61, 107–115. 10.1016/j.etap.2018.05.021 29883902

[B2] Alonso-Peña M. Espinosa-Escudero R. Herraez E. Briz O. Cagigal M. L. Gonzalez-Santiago J. M. (2022). Beneficial effect of ursodeoxycholic acid in patients with acyl-CoA oxidase 2 (ACOX2) deficiency-associated hypertransaminasemia. Hepatology 76 (5), 1259–1274. 10.1002/hep.32517 35395098 PMC9796151

[B3] Bortolini M. Almasio P. Bray G. Budillon G. Coltorti M. Frezza M. (1992). Multicentre Survey of the Prevalence of Intrahepatic Cholestasis in 2520 Consecutive Patients with Newly Diagnosed Chronic Liver Disease. Drug Investig. 4 (4), 83–89. 10.1007/BF03258368

[B4] Cai S. Y. Mennone A. Soroka C. J. Boyer J. L. (2014). All-trans-retinoic acid improves cholestasis in α-naphthylisothiocyanate-treated rats and Mdr2-/- mice. J. Pharmacol. Exp. Ther. 349 (1), 94–98. 10.1124/jpet.113.209353 24492652 PMC3965885

[B5] Cassim Bawa F. N. Xu Y. Gopoju R. Plonski N. M. Shiyab A. Hu S. (2022). Hepatic retinoic acid receptor alpha mediates all-trans retinoic acid's effect on diet-induced hepatosteatosis. Hepatol. Commun. 6 (10), 2665–2675. 10.1002/hep4.2049 35852305 PMC9512485

[B6] Deng F. Qin G. Chen Y. Zhang X. Zhu M. Hou M. (2023). Multi-omics reveals 2-bromo-4,6-dinitroaniline (BDNA)-induced hepatotoxicity and the role of the gut-liver axis in rats. J. Hazard Mater 457, 131760. 10.1016/j.jhazmat.2023.131760 37285786

[B7] Ding L. Zhang B. Zhan C. Yang L. Wang Z. (2014). Danning tablets attenuates α-naphthylisothiocyanate-induced cholestasis by modulating the expression of transporters and metabolic enzymes. BMC Complement. Altern. Med. 14, 249. 10.1186/1472-6882-14-249 25033983 PMC4223591

[B8] Du F. Yao Z. W. (2013). Effect of evodiamine on polycystic ovary syndrome rat. Laser J. 34 (2), 86–88.

[B9] Fickert P. Hirschfield G. M. Denk G. Marschall H. U. Altorjay I. Färkkilä M. (2017). norUrsodeoxycholic acid improves cholestasis in primary sclerosing cholangitis. J. Hepatol. 67 (3), 549–558. 10.1016/j.jhep.2017.05.009 28529147

[B10] Ghonem N. S. Assis D. N. Boyer J. L. (2015). Fibrates and cholestasis. Hepatology 62 (2), 635–643. 10.1002/hep.27744 25678132 PMC4515188

[B11] Hu S. Yin S. Jiang X. Huang D. Shen G. (2009). Melatonin protects against alcoholic liver injury by attenuating oxidative stress, inflammatory response, and apoptosis. Eur. J. Pharmacol. 616 (1-3), 287–292. 10.1016/j.ejphar.2009.06.044 19576882

[B12] Hu Y. Fahmy H. Zjawiony J. K. Davies G. E. (2010). Inhibitory effect and transcriptional impact of berberine and evodiamine on human white preadipocyte differentiation. Fitoterapia 81 (4), 259–268. 10.1016/j.fitote.2009.09.012 19799972

[B13] Ibrahim Z. S. (2015). Chenodeoxycholic acid increases the induction of CYP1A1 in HepG2 and H4IIE cells. Exp. Ther. Med. 10 (5), 1976–1982. 10.3892/etm.2015.2719 26640583 PMC4665948

[B14] Klein K. Winter S. Turpeinen M. Schwab M. Zanger U. M. (2010). Pathway-Targeted Pharmacogenomics of CYP1A2 in Human Liver. Front. Pharmacol. 1, 129. 10.3389/fphar.2010.00129 21918647 PMC3171976

[B15] Kong L. Dong R. Huang K. Wang X. Wang D. Yue N. (2021). Yangonin modulates lipid homeostasis, ameliorates cholestasis and cellular senescence in alcoholic liver disease via activating nuclear receptor FXR. Phytomedicine 90, 153629. 10.1016/j.phymed.2021.153629 34304130

[B16] Labiano I. Agirre-Lizaso A. Olaizola P. Echebarria A. Huici-Izagirre M. Olaizola I. (2022). TREM-2 plays a protective role in cholestasis by acting as a negative regulator of inflammation. J. Hepatol. 77 (4), 991–1004. 10.1016/j.jhep.2022.05.044 35750136

[B17] Li F. (2002). Science of herbal formulas. People's Medical Publishing House.

[B18] Li J. Lian J. (2016). Formulaology. China Press of Traditional Chinese Medicine.

[B19] Liu D. Cao G. Si X. Chen Q. Sun H. (2017). Overview of antiarrhythmic studies on alkaloids in Coptis chinensis. Shandong J. Traditional Chin. 36 (2), 164–166. 10.16295/j.cnki.0257-358x.2017.02.024

[B20] Liu Y. Fu Y. Q. Peng W. J. Yu Y. R. Wu Y. S. Yan H. (2016). Rutaecarpine Reverses the Altered Connexin Expression Pattern Induced by Oxidized Low-density Lipoprotein in Monocytes. J. Cardiovasc Pharmacol. 67 (6), 519–525. 10.1097/fjc.0000000000000372 26859198

[B21] Lu L. Chinese Society of Hepatology and Chinese Medical Association (2022). Guidelines for the Management of Cholestatic Liver Diseases (2021). J. Clin. Transl. Hepatol. 10 (4), 757–769. 10.14218/jcth.2022.00147 36062287 PMC9396310

[B22] Ma B. L. Ma Y. M. (2013). Pharmacokinetic properties, potential herb-drug interactions and acute toxicity of oral Rhizoma coptidis alkaloids. Expert Opin. Drug Metab. Toxicol. 9 (1), 51–61. 10.1517/17425255.2012.722995 22998215

[B23] Martínez-Clemente M. Ferré N. Titos E. Horrillo R. González-Périz A. Morán-Salvador E. (2010). Disruption of the 12/15-lipoxygenase gene (Alox15) protects hyperlipidemic mice from nonalcoholic fatty liver disease. Hepatology 52 (6), 1980–1991. 10.1002/hep.23928 20967760

[B24] Mello A. Hsu M. F. Koike S. Chu B. Cheng J. Yang J. (2021). Soluble Epoxide Hydrolase Hepatic Deficiency Ameliorates Alcohol-Associated Liver Disease. Cell Mol. Gastroenterol. Hepatol. 11 (3), 815–830. 10.1016/j.jcmgh.2020.10.002 33068774 PMC7851189

[B25] Monte M. J. Alonso-Peña M. Briz O. Herraez E. Berasain C. Argemi J. (2017). ACOX2 deficiency: An inborn error of bile acid synthesis identified in an adolescent with persistent hypertransaminasemia. J. Hepatol. 66 (3), 581–588. 10.1016/j.jhep.2016.11.005 27884763

[B26] Natarajan S. K. Stringham B. A. Mohr A. M. Wehrkamp C. J. Lu S. Phillippi M. A. (2017). FoxO3 increases miR-34a to cause palmitate-induced cholangiocyte lipoapoptosis. J. Lipid Res. 58 (5), 866–875. 10.1194/jlr.M071357 28250026 PMC5408604

[B27] Nong C. Zou M. Xue R. Bai L. Liu L. Jiang Z. (2020). The role of invariant natural killer T cells in experimental xenobiotic-induced cholestatic hepatotoxicity. Biomed. Pharmacother. 122, 109579. 10.1016/j.biopha.2019.109579 31794947

[B28] Peng H. (1993). Prescription dictionary of Chinese medicine. People’s Medical Publishing House.

[B29] Qiu J. Yan J. Liu W. Liu X. Lin J. Du Z. (2021). Metabolomics analysis delineates the therapeutic effects of Huangqi decoction and astragalosides on α-naphthylisothiocyanate (ANIT) -induced cholestasis in rats. J. Ethnopharmacol. 268, 113658. 10.1016/j.jep.2020.113658 33307056

[B30] Sangchart P. Panyatip P. Damrongrungruang T. Priprem A. Mahakunakorn P. Puthongking P. (2021). Anti-Inflammatory Comparison of Melatonin and Its Bromobenzoylamide Derivatives in Lipopolysaccharide (LPS)-Induced RAW 264.7 Cells and Croton Oil-Induced Mice Ear Edema. Molecules 26 (14), 4285. 10.3390/molecules26144285 34299559 PMC8304993

[B31] Senoo H. Mezaki Y. Fujiwara M. (2017). The stellate cell system (vitamin A-storing cell system). Anat. Sci. Int. 92 (4), 387–455. 10.1007/s12565-017-0395-9 28299597

[B32] Shen T. (2007). Experimental study on the lipid-lowering effect of HuangLian and Wuzhuyu formula in experimental high-fat model mice. J. Chengdu Univ. Traditional Chin. Med. 30 (1), 18–19. 10.13593/j.cnki.51-1501/r.2007.01.009

[B33] Shen T. Jia B. Guo L. Xu S. (2011). Effects of Huang Lian Wu Zhi Zhu on liver tissue in a rat hyperlipidemia model ABCA 1 gene expression. J. Chengdu Univ. Traditional Chin. Med. 34 (1), 49–51. 10.13593/j.cnki.51-1501/r.2011.01.018

[B34] Sugatani J. Wada T. Osabe M. Yamakawa K. Yoshinari K. Miwa M. (2006). Dietary inulin alleviates hepatic steatosis and xenobiotics-induced liver injury in rats fed a high-fat and high-sucrose diet: association with the suppression of hepatic cytochrome P450 and hepatocyte nuclear factor 4alpha expression. Drug Metab. Dispos. 34 (10), 1677–1687. 10.1124/dmd.106.010645 16815962

[B35] Sun C. Liu W. Lu Z. Li Y. Liu S. Tang Z. (2021). Hepatic miR-378 modulates serum cholesterol levels by regulating hepatic bile acid synthesis. Theranostics 11 (9), 4363–4380. 10.7150/thno.53624 33754066 PMC7977473

[B36] Takitani K. Kishi K. Miyazaki H. Koh M. Tamaki H. Inoue A. (2018). Altered Expression of Retinol Metabolism-Related Genes in an ANIT-Induced Cholestasis Rat Model. Int. J. Mol. Sci. 19 (11), 3337. 10.3390/ijms19113337 30373117 PMC6274878

[B37] Trauner M. Fuchs C. D. (2022). Novel therapeutic targets for cholestatic and fatty liver disease. Gut 71 (1), 194–209. 10.1136/gutjnl-2021-324305 34615727 PMC8666813

[B38] Wang W. (2016). A review on pharmacologic effects of effective ingredients in huanglian. Clin. J. Chin. Med. (8), 147–148. 10.3969j/issn.1674-7860.2016.26.080

[B39] Wang W. Jiang S. Xu C. Tang L. Liang Y. Zhao Y. (2022). Transcriptome and Gut Microbiota Profiling Analysis of ANIT-Induced Cholestasis and the Effects of Da-Huang-Xiao-Shi Decoction Intervention. Microbiol. Spectr. 10 (6), e0324222. 10.1128/spectrum.03242-22 36409145 PMC9769994

[B40] Wu H. Chen C. Ziani S. Nelson L. J. Ávila M. A. Nevzorova Y. A. (2021). Fibrotic Events in the Progression of Cholestatic Liver Disease. Cells 10 (5), 1107. 10.3390/cells10051107 34062960 PMC8147992

[B41] Wu N. Meng F. Zhou T. Han Y. Kennedy L. Venter J. (2017). Prolonged darkness reduces liver fibrosis in a mouse model of primary sclerosing cholangitis by miR-200b down-regulation. Faseb J. 31 (10), 4305–4324. 10.1096/fj.201700097R 28634212 PMC5987749

[B42] Wu P. Chen Y. (2019). Evodiamine ameliorates paclitaxel-induced neuropathic pain by inhibiting inflammation and maintaining mitochondrial anti-oxidant functions. Hum. Cell 32 (3), 251–259. 10.1007/s13577-019-00238-4 30701373

[B43] Xie W. Cao Y. Xu M. Wang J. Zhou C. Yang X. (2017). Prognostic Significance of Elevated Cholestatic Enzymes for Fibrosis and Hepatocellular Carcinoma in Hospital Discharged Chronic Viral Hepatitis Patients. Sci. Rep. 7 (1), 10289. 10.1038/s41598-017-11111-5 28860489 PMC5579038

[B44] Xu D. Qiu C. Wang Y. Qiao T. Cui Y. L. (2021). Intranasal co-delivery of berberine and evodiamine by self-assembled thermosensitive *in-situ* hydrogels for improving depressive disorder. Int. J. Pharm. 603, 120667. 10.1016/j.ijpharm.2021.120667 33933642

[B45] Xu W. Miao m. (2021). Application Analysis of Cholestasis Animal Models Based on Data Mining. Pharmacol. Clin. Chin. Materia Medica. 10.13412/j.cnki.zyyl.20211206.005

[B46] Yan L. Xianzhi L. Hua X. (2016). Effect of adenosine methionine combined with glucocorticoid in the treatment of drug-induced cholestasis liver disease. Chin. J. Difficult Complicat. Cases 15 (2), 176–182. 10.3969/j.issn.1671-6450.2016.02.018

[B47] Yang Z. Meng Y. Wang Q. Yang B. Kuang H. (2011). Property and taste pharmacological evaluation of the chemically resolved fractions of Evodia officinalis. Inf. Traditional Chin. Med. 28 (6), 20–23. 10.19656/j.cnki.1002-2406.2011.05.006

[B48] Yu H. Li Y. Xu Z. Wang D. Shi S. Deng H. (2018). Identification of potential biomarkers in cholestasis and the therapeutic effect of melatonin by metabolomics, multivariate data and pathway analyses. Int. J. Mol. Med. 42 (5), 2515–2526. 10.3892/ijmm.2018.3859 30226547 PMC6192756

[B49] Yu H. Liu C. Wang J. F. Han J. Zhang F. H. Zhou X. (2022). miRNA and miRNA target genes in intervention effect of Zhuyu pill on cholestatic rat model. J. Ethnopharmacol. 283, 114709. 10.1016/j.jep.2021.114709 34626777

[B50] Yu H. Liu C. Zhang F. Wang J. Han J. Zhou X. (2021). Efficacy of Zhuyu Pill Intervention in a Cholestasis Rat Model: Mutual Effects on Fecal Metabolism and Microbial Diversity. Front. Pharmacol. 12, 695035. 10.3389/fphar.2021.695035 34539394 PMC8443775

[B51] Yuan Z. Wang G. Qu J. Wang X. Li K. (2018). 9-cis-retinoic acid elevates MRP3 expression by inhibiting sumoylation of RXRα to alleviate cholestatic liver injury. Biochem. Biophys. Res. Commun. 503 (1), 188–194. 10.1016/j.bbrc.2018.06.001 29885283

[B52] Zhang W. Zhong W. Sun Q. Sun X. Zhou Z. (2017). Hepatic overproduction of 13-HODE due to ALOX15 upregulation contributes to alcohol-induced liver injury in mice. Sci. Rep. 7 (1), 8976. 10.1038/s41598-017-02759-0 28827690 PMC5567196

[B53] Zhang Y. Chen Y. Zhang Z. Tao X. Xu S. Zhang X. (2022a). Acox2 is a regulator of lysine crotonylation that mediates hepatic metabolic homeostasis in mice. Cell Death Dis. 13 (3), 279. 10.1038/s41419-022-04725-9 35351852 PMC8964741

[B54] Zhang Y. Luo J. X. Li Y. G. Fu H. F. Yang F. Hu X. Y. (2022b). An Open-Label Exploratory Clinical Trial Evaluating the Effects of GLS (Coptidis Rhizoma-Evodiae Fructus 2:1) on Fibroblast Growth Factor 21 in Patients with Nonalcoholic Fatty Liver Disease. Evid. Based Complement. Altern. Med. 2022, 4583645. 10.1155/2022/4583645 PMC8967510 35368766

[B55] Zhu B. Zhao L. Liu Y. Jin Y. Feng J. Zhao F. (2019). Induction of phosphatase shatterproof 2 by evodiamine suppresses the proliferation and invasion of human cholangiocarcinoma. Int. J. Biochem. Cell Biol. 108, 98–110. 10.1016/j.biocel.2019.01.012 30682488

[B56] Zordoky B. N. Anwar-Mohamed A. Aboutabl M. E. El-Kadi A. O. (2011). Acute doxorubicin toxicity differentially alters cytochrome P450 expression and arachidonic acid metabolism in rat kidney and liver. Drug Metab. Dispos. 39 (8), 1440–1450. 10.1124/dmd.111.039123 21571947

