# Supplementary material for: The zhuyu pill relieves rat cholestasis by regulating the mRNA expression of lipid and bile metabolism associated genes
Source: Front Pharmacol. 2023 Oct 10;14:1280864. doi: 10.3389/fphar.2023.1280864 (PMC10597705; doi:10.3389/fphar.2023.1280864)
Supplement: Supplementary file 1 [file DataSheet2.DOCX]

***mRNA validation using real-time quantitative qRT-PCR***

**Total RNA was extracted from liver tissue using the mirVanaTM RNA Isolation Kit, AM1561, according to the manufacturer’s speciﬁcations. RNA yield was determined using a NanoDrop 2000 spectrophotometer (Thermo Scientific, USA), and the integrity was evaluated by agarose gel electrophoresis stained with ethidium bromide. Quantiﬁcation was performed using a two-step reaction process: reverse transcription (RT) and PCR. Each RT reaction consisted of 0.5 μg RNA, 2 μl 5x TransScript All-in-one Super Mix for qPCR, and 0.5μl gDNA Remover, in a total volume of 10 μl. Reactions were performed in a Gene Amp^®^ PCR System 9700 (Applied Biosystems, USA) for 15 min at 42℃ and 5 s at 85℃. The 10 μl RT reaction mix was then diluted 10x in nuclease-free water and stored at -20℃.**

**qRT-PCR was performed using a Light Cycler^®^ 480 Ⅱ Real-time PCR Instrument (Roche, Swiss) with 10 μl PCR reaction mixture including 1 μl cDNA, 5 μl 2x PerfectStartTM Green qPCR SuperMix, 0.2 μl forward primer, 0.2 μl reverse primer and 3.6 μl nuclease-free water. Reactions were incubated in a 384-well optical plate (Roche, Swiss) at 94 ℃ for 30 s, followed by 45 cycles of 94 ℃ for 5 s and 60 ℃ for 30 s. Each sample was run in triplicate. At the end of the PCR cycles, a melting curve analysis was performed to validate the speciﬁc generation of the expected PCR product. The expression levels of the mRNAs were normalized to ACTB and calculated using the 2^-ΔΔCt^ method (Livak & Schmittgen, 2001).**
