# Supplementary material for: The zhuyu pill relieves rat cholestasis by regulating the mRNA expression of lipid and bile metabolism associated genes
Source: Front Pharmacol. 2023 Oct 10;14:1280864. doi: 10.3389/fphar.2023.1280864 (PMC10597705; doi:10.3389/fphar.2023.1280864)
Supplement: Supplementary file 2 [file DataSheet1.DOCX]

*ZYP Preparation and Quality Control*

In this study, the ZYP decoction was prepared by boiling the herbs in water twice. For every 240 ml of water, there were 6 g each *of Coptis chinensis* Franch. and *Tetradium ruticarpum* (A. Jussieu) T. G. Hartley. The mixture was heated at 100°C for 45 min, filtered, and the suspension was collected for later use. The filtered crude herbs were then added to another 240 ml of water, heated at 100°C for 45 min, filtered, and the suspension was collected. The two suspensions were mixed thoroughly and concentrated to 120 ml using a rotary evaporator. As a result, 120 ml of solution was extracted from 12 g of crude Chinese herbs, making the final concentration of ZYP 0.1 g/ml (w/v). The extraction solution was stored at -20°C before administration.


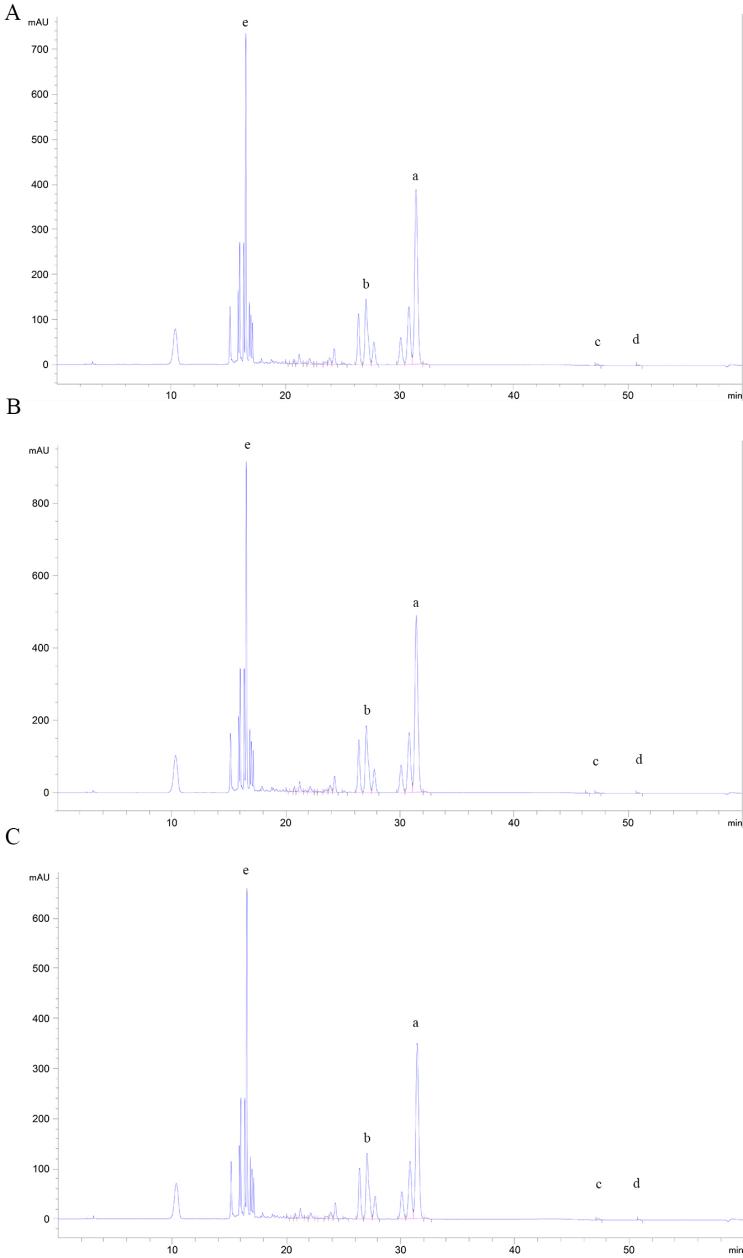


**Fingerprint of the Zhuyu pill and its formulae.** (A-C) Three ZYP technical repetitions in HPLC analyses. a) berberine; b) coptisine; c) evodiamine; d) rutaecarpin; e) other unknown materials.
